# Optimizing language for effective communication of gene therapy concepts with hemophilia patients: a qualitative study

**DOI:** 10.1186/s13023-020-01555-w

**Published:** 2021-04-28

**Authors:** Daniel P. Hart, Brian R. Branchford, Sarah Hendry, Robert Ledniczky, Robert F. Sidonio, Claude Négrier, Michelle Kim, Michelle Rice, Matthew Minshall, Claire Arcé, Steve Prince, Maria Kelleher, Sharon Lee

**Affiliations:** 1 Royal London Haemophilia Centre, Barts and The London School of Medicine and Dentistry, Queen Mary University London, London, E1 1BB UK; 2 Hemophilia and Thrombosis Center, University of Colorado School of Medicine and Children’s Hospital Colorado, Aurora, CO USA; 3 Maslansky + Partners, 200 Varick Street, Suite 601, New York, NY 10014 USA; 4 Aflac Cancer and Blood Disorders Center, Atlanta, GA USA; 5 Hemophilia and Thrombosis Center, Hôpital Cardiologique, Université Lyon 1, Lyon, France; 6 Hemophilia Foundation of Southern California, Pasadena, CA USA; 7 National Hemophilia Foundation, New York, NY USA; 8 The Haemophilia Society, London, UK; 9 Association française des hémophiles, Paris, France; 10 BioMarin Pharmaceutical Inc., Novato, CA USA

**Keywords:** Gene therapy, Gene transfer, Adeno-associated virus (AAV), Hemophilia A, Vocabulary, Language, Lexicon, Communication, Consensus, Qualitative research

## Abstract

**Background:**

For communities of people living with hemophilia and other genetic conditions, gene therapy could represent a paradigm shift in treatment strategies. As investigational therapeutic modalities such as gene therapy become more widely used and discussed, there is a critical need for all stakeholders to communicate using a lexicon that is intelligible, accurate, consistent, and representative of novel treatments. In doing so, expectations can be more carefully managed and potential risks, benefits, and limitations better understood. In recognition of this need, a first-ever study of gene therapy lexicon was conducted using established methods of market research and linguistic analysis.

**Methods:**

Ninety-four participants representing hematologists, nurses, caregivers, and people with hemophilia A, in six countries (US, UK, Spain, Germany, France, Italy) took part in a series of in-depth interviews, face-to-face focus groups, an advisory board meeting, and online group interviews to develop, refine, and test verbal, written, and pictorial language concepts through a three-phase iterative process. Sessions were conducted in local languages using detailed discussion guides. Feedback from participants was captured using real-time instant-response dial testing to measure moment-by-moment emotional responses to language stimuli. Semiquantitative analysis of the responses informed selection of preferred language concepts for final testing, and qualitative discussion explored preference rationale. Participants also completed polling and forced rank and choice written exercises.

**Results:**

Study feedback showed that the hemophilia community has preferences around consistent lexicon to describe hemophilia and its management. Expert linguistic analysis of feedback from the three phases enabled agreement of a consensus lexicon of vocabulary and an optimized summary narrative for talking about gene therapy amongst people affected by hemophilia A. Preferences were largely consistent across audiences and countries, although some country-specific recommendations were made. A representative summary phrase was agreed: “Gene therapy is being studied in clinical trials with the aim to allow the body to produce factor VIII protein on its own”.

**Conclusions:**

The use of preferred language across different stakeholders increases understanding and comfort during discussions of novel and complex therapeutic modalities such as gene therapy. Consistent use of community-informed lexicon minimizes miscommunication and facilitates informed decision-making regarding potential future treatment opportunities.

## Background

For communities of people living with hemophilia and other genetic conditions, the approval of a gene therapy could represent a paradigm shift in the rapidly evolving succession of novel therapeutic strategies. One of the challenges associated with developing any potential new therapy is to ensure that all involved parties (patients, physicians, patient advocates, nurses, caregivers, reimbursement agencies, drug developers, and regulators) have and understand the information they need to make informed decisions about the investigational product’s safety, efficacy, risks, benefits, and appropriateness for each individual. As treatment modalities become increasingly complex, there is a critical need for stakeholders to communicate amongst one another with a lexicon that is intelligible, accurate, consistent, and representative of emerging new therapeutic strategies such as gene therapy. In doing so, expectations can be more carefully managed; potential risks, benefits, and limitations can be better understood; and research findings can be shared with the greatest possible levels of transparency.

Hemophilia A and B are X-linked monogenic inherited disorders caused by mutations in the *F8* and *F9* genes encoding factor VIII and factor IX proteins, respectively, leading to a partial or complete absence of the corresponding endogenous clotting factors. Current standard of care with prophylactic factor replacement therapy requires life-long regular intravenous infusions several times a week, which presents a substantial treatment burden for patients and high costs for healthcare systems [1–3]. Extended half-life products have become available more recently, which may enable reduced dosing frequency or improve protection from bleeding events by maintaining higher trough levels of clotting factors [4], but real-world evidence suggests that these regimens could result in higher drug costs for patients and healthcare systems [5, 6]. Novel non-factor therapeutic agents in development, which act by enhancing coagulation or inhibiting anticoagulant pathways, also aim to provide less burdensome, longer-acting prophylaxis and have demonstrated promising hemostatic properties in clinical trials [7]. Among these, emicizumab (Hemlibra) is indicated for routine prophylaxis in people with hemophilia A and offers a comparable level of protection to factor VIII prophylaxis in an extended regular subcutaneous dosing interval of 1 to 4 weeks [8]. It does not, however, eliminate the need for FVIII clotting factor treatment for breakthrough bleeds and surgery. Thus, in spite of these advances, there remains a need for less burdensome and more cost-effective treatment that limits the multiple long-term complications that people with hemophilia continue to suffer [9]. With their monogenic etiology, hemophilia A and B are, therefore, ideal candidates for gene therapy.

Technological advances and better understanding of therapeutic viral vectors have led to the development of non-pathogenic, tissue-targeted candidate gene transfer therapies for a range of monogenic diseases [10]. After decades of research and some major challenges overcome, rapid progress is now being realized with the use of adeno-associated virus (AAV)-mediated gene therapy in hemophilia [11]. The first successful gene transfers in this field were in people with hemophilia B, where single infusions of AAV expressing optimized human *F9* transgenes have produced sustained therapeutic expression of factor IX coagulant activity [12–14], with longer-term follow-up demonstrating sustained activity for up to 8 years at the time of reporting [15].

The potential for long-term correction of factor VIII deficiency in hemophilia A with AAV-mediated gene therapy is also becoming a reality. Technological challenges relating to the large size of the *F8* gene and inefficient expression of the human factor VIII coding sequence have been overcome with the development of a codon-optimized B-domain-deleted human *F8* gene construct that can be delivered successfully by AAV [16]; and there is some preclinical evidence in both hemophilia A and hemophilia B to support the possibility that immunogenicity to transgene products can be overcome by directing gene transfer vectors to the liver to induce tolerance to preexisting factor inhibitors [17, 18]. Valoctocogene roxaparvovec is an AAV5-mediated gene therapy undergoing clinical trials for the treatment of hemophilia A; it delivers a functional, codon-optimized, B-domain-deleted, human *F8* gene under the control of a liver-specific promoter (AAV5-hFVIII-SQ). In an ongoing Phase 1/2 clinical trial, a single intravenous infusion of valoctocogene roxaparvovec at a dose of 6 × 10^13^ vector genomes (vg) per kilogram (kg) demonstrated sustained reductions in annualized bleed rate, resolution of target joints, and cessation of prophylactic factor VIII replacement therapy in men with severe hemophilia A [19], with effects lasting up to 3 years at the time of reporting [20].

Patients with chronic illnesses are becoming more educated and empowered to advocate for their own care and be actively involved in shared decision making about treatment [21, 22]. Hemophilia is no exception [23]; many people with hemophilia recognize the need to continue to grow in their understanding and active management of their disease in order to minimize risk of bleeds and injuries, to recognize and assess the severity of bleeding events when they occur, and to know when to self-administer or seek medical intervention. Within the rapidly evolving hemophilia treatment landscape, patients need to be actively engaged in seeking and learning about new treatment options that will improve their day-to-day quality of life and long-term health status [9, 23].

With gene therapy increasingly being viewed as a viable treatment option for hemophilia, it is important that an agreed lexicon embraces the complex concepts and considerations around this novel potential treatment modality. Communications need to be delivered in a way that respects and acknowledges differences in each person’s relationship with hemophilia and background in the scientific or medical fields, and presents gene therapy using accurate, plain, and consistent language [9, 24]. Indeed, many scientific journals now include a plain language summary to accompany scientific journal articles, in addition to the usual publication abstract, so that non-specialists can quickly and easily understand key findings.

To our knowledge, the language of gene therapy has not previously been explored with hemophilia patients and those involved in their care. A qualitative study of gene therapy lexicon was conducted to identify the most appropriate language with which to effectively communicate information about AAV-based gene therapy among healthcare professionals (HCPs), patient advocates, caregivers, and patients affected by hemophilia A. The aim was to develop a preferred vocabulary and optimized summary narratives addressing the key concepts “What is gene therapy?”, “What causes hemophilia?”, and “How gene therapy works”.

## Methods

### Study design and setting

The study utilized a three-part cumulative approach involving a series of in-depth telephone interviews, face-to-face focus groups, an advisory board meeting, and online group interviews conducted between June 2018 and September 2018. Participants represented hematologists, nurses, caregivers, patients, and patient advocates in the US and in five European countries: UK, Spain, Germany, France, and Italy. Detailed discussion guides were used to collect, refine, and test language and image concepts. Instant-response methodology was used to capture participants’ preferences and emotional responses to specific language concepts and examples. An iterative process was used in which the initial interview guides were updated and language concepts refined as more input was gained from responders. Country-specific sessions were conducted in local languages by independent research professionals contracted by the study team. All phases of the study were designed and implemented by market research professionals specialized and highly experienced in language research strategies and focus group methodologies. The team utilized their linguistic expertise to interpret the outputs at each phase of the study to inform selection and refinement of the final lexicon recommendations.

### Participant selection

Subjects potentially willing and suitable for participation in the study were identified from market research panels and/or through existing research relationships with the study sponsor. Participants in Phase I—which shaped the preliminary language framework for the study—included some of our expert hematologists, because of their deep knowledge of gene therapy through their involvement and experience in clinical trials. Conversely, participants in the study roll-out Phases II and III were intended to be more representative of the general hemophilia community; structured screener interviews were used to confirm eligibility based on criteria detailed in Table 1, and ensure a diverse set of participants, representing a range of countries (US, UK, Spain, Germany, France, and Italy) and audiences (hematologists, nurses, patient advocates, caregivers, and patients).

**Table 1:** Eligibility criteria for subject participation in the study

Hematologists	Nurses
• Primary specialty in either hematology or hematology/oncology	• Qualified as a Registered Nurse or Nurse Practitioner
• Board certified/eligible (applies to US physicians only)	• Actively involved in the management of hemophilia
• Has been in practice for 2–35 years	• Has been in practice for between 4 and 30 years
• Spends a minimum 50% of time in direct patient contact	• Spends a minimum 75% of time in direct patient contact
• More than 25% of patients are moderate^a^/severe^b^ hemophilia patients	
• 50% of patients must receive prophylactic treatment with factor VIII	
**Hematologists and nurses**	
• Is involved in the care of 10 or more hemophilia A patients (in US; 5 or more in other countries)	
• Spends more than 20% of work time in a hemophilia treatment/care center	
**Target quotas**	
At least 50% of hematologists and nurses to be involved predominantly in treating adult patients. No more than 2 hematologists to be involved in a clinical trial of gene therapy or emicizumab	

Patients	Caregivers
• Age 18 to 60 years	• Age 18 to 60 years
• Diagnosis of hemophilia A	• Is primary caregiver of someone diagnosed with hemophilia A
• Must be on prophylactic standard or extended half-life factor VIII	• Is caring for someone on prophylactic standard or extended half-life factor VIII^c^
	• Is not a professional caregiver
	• Has not been a physician, physician’s assistant, pharmacist, mental health professional, nurse practitioner, or registered/licensed nurse
Age distribution to be evenly split between 18–30, and 31–60 years	Aimed for 75% caregivers of severe hemophilia^*b*^/ 25% moderate hemophilia^a^
No more than 1(2) US (EU5) participants with factor VIII inhibitors	No more than 1 participant caring for someone who has developed Factor VIII inhibitors
**Target quotas**	
No more than 1 patient and 1 caregiver of a patient per country enrolled in emicizumab trial. No more than 10% of patients and caregivers to be highly involved in hemophilia patient groups (as organizer, advocate, or speaker); no more than 30% of patients to be regular attendees of patient group activities	

*All participants*	
• Does not (and close family members do not) have a relationship^d^ with any of the following types of companies - Medical equipment manufacturer - Market research or advertising firm - Marketing or healthcare consulting firm - Local, state, or federal government • Has not participated in market research in the past 12 months	

^a^Moderate defined as factor VIII activity between 1 IU/dL and 5 IU/dL (normal range 50–150 IU/dL); bleeds occur upon injury; occasional breakthrough/spontaneous bleeds; treatment can be on-demand or prophylactic

^b^Severe defined as factor VIII activity less than 1 IU/dL; bleeds occur upon injury; generally have frequent breakthrough/spontaneous bleeds without treatment (once a month or more); on prophylactic treatment to control bleeds

^c^An immediate family member or other non-professional caregiver (e.g. friend/neighbor)

^d^Works for/consults with/serves on the advisory board of/holds any ownership position (excluding stock)

Hematologists and nurses were actively involved in managing hemophilia patients (defined as comprising at least 20% of their working hours). Caregivers were primary non-professional caregivers to an individual with severe hemophilia A. Patients were 18–60 years old with a diagnosis of hemophilia A and receiving prophylactic factor VIII, representing those with severe disease and a population similar to that in which gene therapy clinical trials have been conducted. Enrollment criteria specified limits on the proportions of hematologists already actively involved with gene therapy or novel treatments and on patients’ experience of novel treatments or active involvement in hemophilia patient groups (see Table 1).

### Study procedures

**Fig. 1:**
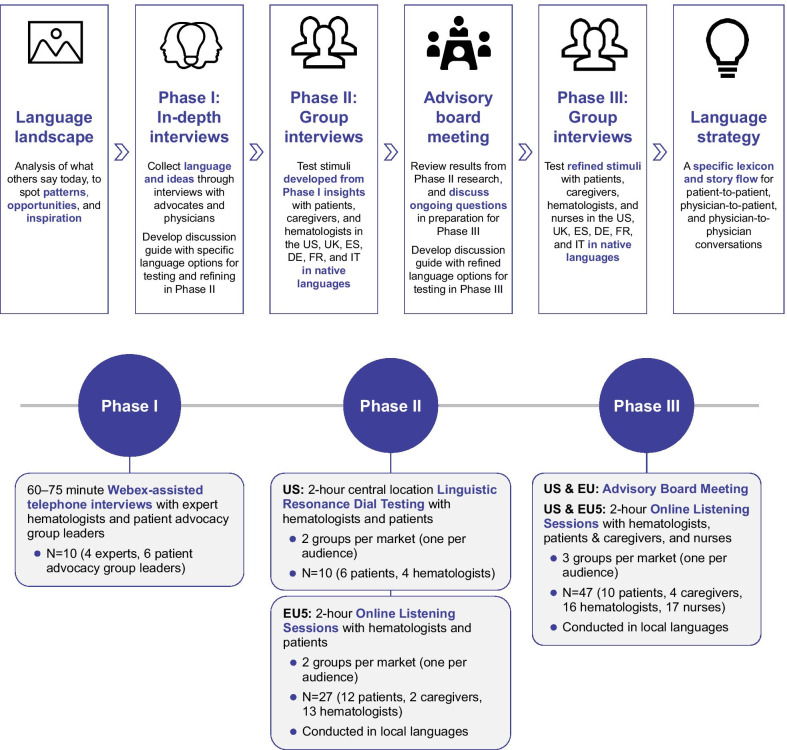
Study design

The study was conducted in a series of steps including background research, in-depth interviews (Phase I), face-to face focus groups per audience per country (Phase II), an advisory board meeting to consolidate findings, and a final round of online group interviews to test derived concepts (Phase III) (Fig. 1).

In Phase I, in-depth interviews were conducted with four expert hematologists and six patient advocacy group leaders to discuss findings of background language research and to shape the preliminary language concepts to test with participants in subsequent phases of the study. These were 60- to 75-min Webex-assisted telephone interviews led by market research professionals who are specialists in language strategy; all of these interviews were conducted in English. Feedback from the Phase I interviews was used to develop a comprehensive Discussion Guide for use in Phase II, designed to ensure a consistent approach across the study groups. Language concepts included in the Phase II Discussion Guide comprised short, distinct phrases and descriptors representing different aspects of gene therapy under three main themes: *1. Mechanism of disease/What is a gene? 2. What is gene therapy? 3. How does gene therapy work?* Content was relevant to the potential application of gene therapy in the treatment of hemophilia A and was prioritized such that concepts could be reviewed and key questions answered within a 120-min interview. Two versions of the Phase II Discussion Guide were developed, aimed at hematologists and patients/caregivers, respectively.

In Phase II, concepts developed from Phase I insights were explored in detail in group sessions of two to three participants. Two focus groups were held in each country—one for hematologists and one for patients/caregivers—and each 2-h session was led by an independent moderator, who followed the structured workflow and decision-making process defined in the respective Phase II Discussion Guide. Feedback on pictorial, written, and verbal stimuli was solicited via a combination of written exercises, group discussion, and instant-response testing, with planned prompts and questioning led by the moderator. Participants were encouraged to share their own personal opinions, whether positive or negative, and not to be influenced by what they believed others might think or might want to hear.

Feedback from the Phase II focus groups was used to refine descriptors and expressions that are most meaningful to the respective target audience. An advisory board meeting attended by three hematologists and 13 patient advocacy group leaders—including those involved in shaping the preliminary language concepts in Phase I—was held to review the results and discuss ongoing questions. The Phase II Discussion Guide was updated with outputs of the discussions and refined language concepts to develop a Phase III Discussion Guide for testing with a larger audience in the Phase III video interviews/focus groups. The participants in Phase III interviews were intentionally naïve to language concepts developed in earlier phases of the study and did not include subjects who had participated in Phase II. The intention was to ensure that as the lexicon developed, the final lexicon would be clearly understood by those who had not been exposed to it previously. The target audience groups in Phase III were also expanded to include nurses.

### Evolution of gene therapy lexicon

Across multiple topics relating to gene therapy as a potential treatment for hemophilia, images and descriptive statements were presented, discussed, examined, and refined. The baseline language for discussion was established with input from the key experts interviewed during Phase I of the study and is detailed in Additional File 1. Thereafter, in Phase II and Phase III, the moderator in each focus group led discussions through a series of predefined language stimuli. Using a variety of feedback techniques, the preferred words, phrases, and pictorial representations were modified and agreed upon through an iterative and adaptive process. Conversely, undesirable, disagreeable, or confusing language was removed from the narrative.

After some refinement of messages and supporting images following analysis of feedback from Phase II, Phase III focus groups reviewed five derived story flow options to agree on an optimal sequence and level of detail for the gene therapy narrative. Some additional testing and selection of key descriptors was also performed. Materials reviewed in Phase III are detailed in Additional File 2.

### Analyses

**Fig. 2:**
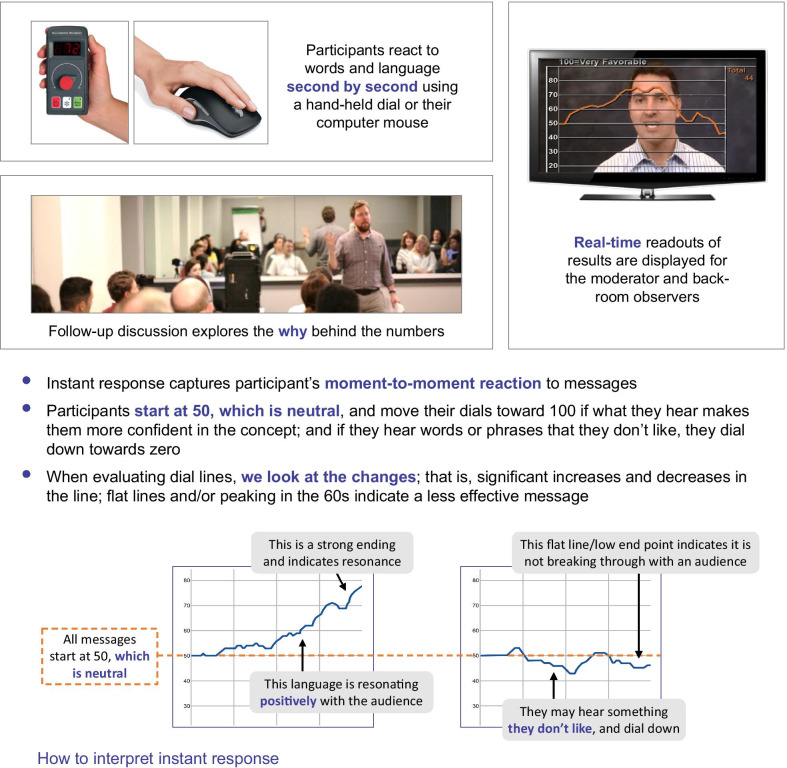
Phase II: Linguistic resonance dial testing

For Phase II focus groups conducted face-to-face (i.e. those in the US), feedback from participants was captured in real time using Linguistic Resonance Dial Testing, a proprietary technology for implementing instant-response methodology (Fig. 2) [25]. This technology enables instant word-by-word measurement of emotional responses to a wide variety of language stimuli, to select and refine the phrases that resonate best with the target audience. During each session, participants reacted to the language stimuli on a moment-to-moment basis, using a dial with a rating of 0 (don’t like) to 100 (like) to continuously rate each message based on their ‘gut feelings’. For each message, the dial was centered at 50 (i.e. neutral), and participants were encouraged to keep their hand on the dial at all times and to react immediately to each word, phrase, or sentence rather than wait to give it a score at the end, using the whole range of the dial to show how they were feeling throughout the delivery of the message. For messages that participants liked (made sense, was clear, was compelling), they turned the dial up towards 100; for something they did not like for any reason, they turned the dial down towards 0. Perception Analyzer 9.0 software was used for collection and analysis of dial responses and was critical to understanding the most promising language to carry into Phase III [25]; this software performs quantitative analyses of the graphical outputs from Linguistic Resonance Dial Testing, with positive lines indicating promising language to use, and negative lines indicating language to avoid. The level and slope of these lines provide a semiquantitative measure of the strength of emotion. Data from the dial testing exercises were collected anonymously.

The quantitative analysis was supplemented by qualitative group discussion to explore the reasoning behind their reactions to particular words, phrases, ideas, or images. Participants also completed written exercises asking them to compare and rank messages and choose the language that was most resonant to them. Back-room viewing of the focus group activities and outputs enabled the assessment team to observe, capture, and analyze responses unseen.

European Phase II focus groups were conducted in native/local language by local teams, with logistics managed remotely by the US-based research team. For this reason, it was not possible to utilize the Linguistic Resonance Dial Testing technology in these sessions and an online focus group format was more practical, using the interactive platform InterVu provided by FocusVision [26]. Similar qualitative discussions and written exercises were performed as those used in the US groups. The European group sessions were conducted in their local languages, with simultaneous translation available so that the assessment team could follow proceedings in real time.

Similar to the qualitative assessments in Phase II, results from Phase III focus groups were derived from qualitative discussion using polling of specific language concepts to identify the most promising terms, as well as verbal forced ranking and choice exercises.

Results of this research are reported descriptively; no statistical analyses were performed.

## Results

### Participants

Experts involved in defining the study protocol and preliminary language concepts for testing included four hematologists and six patient advocacy group representatives. Expert hematologists represented US, UK, and France. Patient advocacy group representatives were from US, UK, France, and Germany (patient advocacy groups from Italy and Spain were contacted, but no response was received). A total of 84 additional subjects met the eligibility criteria and took part in Phase II (n = 37) and Phase III (n = 47) of the study (Table 2). Among a total of 28 people with hemophilia, 25 (89%) had severe hemophilia A and 3 were classified as moderate; none had mild disease. All patients required prophylactic factor replacement therapy and none were on emicizumab or had undergone gene therapy. Overall, 12% (4/34) of patients and caregivers were highly engaged in hemophilia patient groups (as organizer, advocate, or speaker); 18% of patients regularly attended patient group activities.

**Table 2:** Study participants represented four audiences and six countries

	Patients	Caregivers	Hematologists	Nurses	Total
**Phase II**	**18**	**2**	**17**		**37**
United States	6	0	4		10
United Kingdom	3	0	3		6
Spain	3	0	3		6
Germany	1	1	1		3
France	2	1	3		6
Italy	3	0	3		6
**Phase III**	**10**	**4**	**16**	**17**	**47**
United States	2	1	2	3	8
United Kingdom	2	0	3	3	8
Spain	1	1	2	3	7
Germany	2	1	3	3	9
France	2	0	3	2	7
Italy	1	1	3	3	8

Feedback from recruitment interviews demonstrated that, as expected, the study population was generally well informed; patients and caregivers were well educated about the condition and its treatment and HCPs were already knowledgeable about the condition and about gene therapy. This information was helpful in designing materials with the required level of detail for use in focus group discussions. The feedback indicated that all stakeholders are highly engaged in conversations and activities relating to hemophilia and its management—for patients and caregivers, this is their life; and for HCPs it is their life’s work. It was also important to recognize that their experiences, hopes, and expectations are highly individual—where the opportunity of gene therapy can mean different things to different individuals. For example, some patients want to know how gene therapy might impact their current treatment, while others are more interested in understanding if gene therapy could allow them to do more and be more active.

### Feedback from interviews and focus groups

As described in the "Methods", the baseline language set for evaluation was established with input from the key experts interviewed during Phase I of the study and is detailed in Additional File 1. The feedback from focus group sessions and the advisory board in Phase III, and the resulting language recommendations derived from linguistic analysis of the results are presented below by language theme.

1. What is gene therapy?

Understanding of, and preferences around, language for a gene therapy were explored through written feedback and discussion around labelled illustrations and written exercises.

In general terms, describing gene therapy as a “method of treatment” was a positive description suggesting potential differentiation, and was better accepted than alternatives such as treatment approach, mode of treatment, scientific technique, form of treatment, or medical approach. One HCP commented: “*Therapy is not a scientific technique … A scientific technique makes me think of landing on the Moon.*”

In qualifying “method of treatment”, it was important to balance emphasis on the novelty of gene therapy over factor replacement while also controlling expectations about who will benefit. “Novel” was a more inspirational adjective, emphasizing the step change that gene therapy represents, while “potential” was more informative and realistic, reflecting that it may not be suitable or available for everyone. Both “novel” and “potential” were accepted over “revolutionary”, which was perceived as inaccurate, exaggerated, and potentially overpromising: “*Revolutionary means that it is the first of its kind in any disorder. We are already using it in sickle cell, *etc*. Novel … is more realistic.*”

In the context of broader treatment options, people with hemophilia A are knowledgeable about currently available treatments. It is better to talk about “what gene therapy is” rather than what current treatments “are not”. They do not need to have the difference between gene therapy and traditional factor replacement therapy explained. However, an important differentiating feature is its administration via a single, one-time IV infusion: "*I inject all the time, constantly inserting factor all the time. Take something just once and the body takes over and produces the factor—I like that idea.*”

Turning to gene therapy specifically, descriptions of the multiple different types of gene therapy under development were found to be confusing and overwhelming to some patients and they favored focusing the discussion solely on gene transfer. Among a choice of several possible descriptors, the term “AAV gene transfer” emerged as that which most accurately and simply described this type of therapy (Table 3). HCPs agreed that there was no need to talk about other types of gene therapy, and the proposed image of different types of gene therapy (gene transfer versus gene editing versus cell therapy) (see Additional File 1) was considered irrelevant in the present context.

**Table 3:**
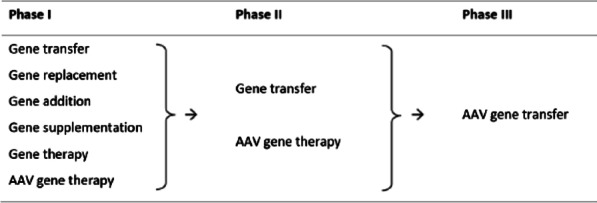
Derivation through sequential project phases of the preferred term “AAV gene transfer” (type of therapy)

Finally, for talking about the developmental status of gene therapy, “undergoing clinical trials” was the preferred approach—acknowledging that approval is still needed but indicating that it is approaching the last stage of testing, while not overpromising. In contrast, “Under clinical investigation” could be interpreted as signaling a problem that needs to be explored, while “in development” suggested much earlier development, before clinical trials and a long way off potential availability to patients.

2. Mechanism of disease

Moderated discussions and language selection exercises quickly revealed that people with hemophilia A are informed and educated about their condition and that their knowledge is enough for them to understand genetics and the potential for gene therapy. There is no need to educate up front on what a gene is. However, there are some key language considerations for talking about hemophilia A and the mechanism of disease, especially when following up with more detail. For this theme, linguistic resonance testing using the instant-response dial technology was employed.

An important message was that one can demonstrate more empathy when talking directly to individuals with hemophilia by describing them as “people with hemophilia A”, not just patients—they are not defined by their disease: “*Medically speaking, I’m a patient, but talk about ‘people with hemophilia A’—it’s more human.*” However, for HCPs, “patients” aligns more closely with their day-to-day experience and is the accepted term: “*If you are talking to medical professionals, you think of your ‘patients’.*”

Describing the condition of hemophilia A, labeling hemophilia as a “disease” was often met with an immensely emotional response from patients: “*Diseases are associated with such horrible things, that’s not how I think about hemophilia*”. The term “condition” was used most consistently by patients across countries; “disorder” (e.g. bleeding disorder) was often used by advocacy organizations.

Surprisingly to most HCPs, patients are generally comfortable with the term “mutation” (i.e. genetic mutation). HCPs across markets were hesitant to use the word “mutation”, because they were concerned that patients will react negatively: “*Mutation can have a negative connotation. Mutation makes it sound like a mutant. Probably better to ‘sugarcoat’ it a little bit*”. Given a choice of alternative descriptors, “variation” or “change” were perceived by patients as terms that did not imply anything wrong; “defect”, “hiccup”, and “mistake” were considered more negative. However, to most patients, “mutation” meant nothing more than how the disease is defined and was considered the most helpful description: “*Mutation is better. It’s a gene that changes.*” Balancing perceived accuracy and emotional response, “mutation” stood out as the best term to use across countries.

The function of genes was most appropriately described by the term “step-by-step instructions”. This was considered a familiar term whilst being prescriptive enough to clearly describe the role of a gene. Other options were either seen as too inflexible (“computer code”), too variable (“recipe”), or did not translate well across some countries (“blueprint”).

When evaluating images, participants requested more emphasis on the gene, and less emphasis on the cell and its components, as the gene is most relevant to hemophilia. Visuals that illustrate the scientific connection between genes and proteins (clotting factors) were considered helpful in explaining the mechanism of disease.

3. How is gene therapy designed to work?

The role of the viral vector in gene transfer therapy is a key concept to understand. A combination of instant-response dial testing, moderated discussion, and written language selection exercises were employed in this section. Although patients might be expected to be alarmed about the use of a virus in gene therapy, a fear that was echoed by some physicians, feedback from patients indicated that talking about the role of AAV upfront is the best way to gain their trust and understanding of the product: “*Not all viruses are bad. You may need to explain that it’s not known to cause sickness in people…*”*.* If the virus is not discussed until later, the listener may suspect that key information is being hidden.

However, the right introduction to the virus is essential. Talking about a “neutralized” virus struck the right balance: it emphasized its carrier status and was better received than “nonpathogenic” (too scientific), “non-illness causing” (too simplistic), or “harmless” (not accurate). For patients, “neutralized” was considered a candid description, while also addressing fears over safety. “*The word neutralized would indicate this virus is really just a protein, a carrier—nothing to worry about.*” For HCPs, “nonpathogenic” virus was preferred as a more technical term that was more familiar to doctors and nurses.

Describing the role of the virus, “viral shell” was a more helpful description than words such as “envelope”, “carrier”, “capsid/capsule”, or “polyhedron”. With “viral shell” it was easy to visualize the healthy gene being placed inside and it implied it is broken down once the gene is delivered—it clearly explained the role of the virus in a way that patients were able to quickly understand. However, it did not convey the idea of the gene being transported, where “vehicle” was considered a more suitable term to represent the role the virus plays in delivering the healthy gene. The new gene inside the viral shell, or vehicle, was best described as a “functional gene”. Functional differentiates the new gene without degrading the patient’s existing genes. “Healthy gene” did not work in this context as it suggested that other genes are unhealthy.

In terms of how gene therapy works, the concept of “targeting” the gene responsible for creating factor VIII was well understood. However, this leads to the question “*where does the infusion go?*” Current gene therapies in development for hemophilia A target the liver. For both patients and HCPs alike, it was important to explain the role the liver plays. For patients, they wanted to know where this method of treatment works in the body, while physicians felt this information is important for determining who may or may not be a candidate for gene transfer.

It is important to clarify what happens to patients’ existing genes when they receive AAV gene transfer therapy. Talking about how the new, functional gene works alongside the existing genes was expected to provide a clear and safe message but this was not well received: “*It replaces the functionality, it does not work alongside it. It allows for the production of what we are missing, but nothing is done with the other gene.*”

**Fig. 3:**
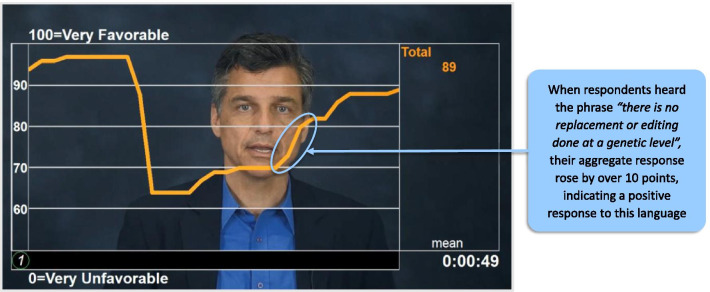
Interpreting responses to linguistic resonance dial testing, illustrating responses to descriptions of gene transfer and gene editing

Patients can also be confused about the difference between gene transfer and gene editing, so it was important to explain that there is no replacement or editing done at a genetic level*—*just the introduction of a new, functional *F8* gene into the body. The key message they wanted to hear more of was that gene transfer leaves the existing genes alone (Fig. 3). This clearly and succinctly distinguishes AAV gene transfer from gene editing.

Overwhelmingly, audiences reported that it is important to know that the new, functional gene cannot be passed down to future generations: “*When my wife and I start a family, there’s a 100% chance that my daughter will be a carrier. It’s important to know that this treatment starts and ends with you, the person using it.*”

### Story flow narrative choices

The initial story flow was based on the null hypothesis that a sequential narrative, moving from more general to more specific information about gene therapy, would be preferred by patients. In Phase II, each section was evaluated in isolation in order to assess the relative importance of each, with verbal feedback collected on the optimal story flow for a potential narrative. The different story flow options used for discussion in the focus groups are detailed in Additional File 2. In Phase III, this alternative narrative story flow (below) was presented against the null hypothesis, with all patients expressing a preference for the alternative, optimized narrative detailed below.

### Agreed summary narrative and vocabulary

The optimized language and narrative to support communication of gene therapy concepts among the hemophilia community, as derived from participant feedback, are summarized in four outputs:

1. Consensus on words and phrases to use and not to use (Table 4)
2. Optimized gene therapy narrative (key summary statements providing a clear description of each of the three themes): “*What is gene therapy?* Gene therapy is a novel method of treatment currently undergoing clinical trials for a variety of genetic conditions, including hemophilia A.* Mechanism of disease*. Because of a genetic mutation, people with hemophilia A don’t produce enough of the factor VIII protein necessary to form stable clots in their blood. The type of gene therapy for hemophilia A is called adeno-associated virus (AAV) gene transfer. AAV gene transfer targets the gene responsible for creating factor VIII.* How gene therapy works*. In AAV gene transfer, a functional gene is inserted into a neutralized viral shell, which delivers the new gene into the liver via a single IV infusion. There is no replacement or editing done at a genetic level*—*just the introduction of a new, functional factor VIII gene into the body, which is not passed down to future generations.”
3. Pictorial representation of gene therapy and how it works in the body (Fig. 4).

**Table 4:**
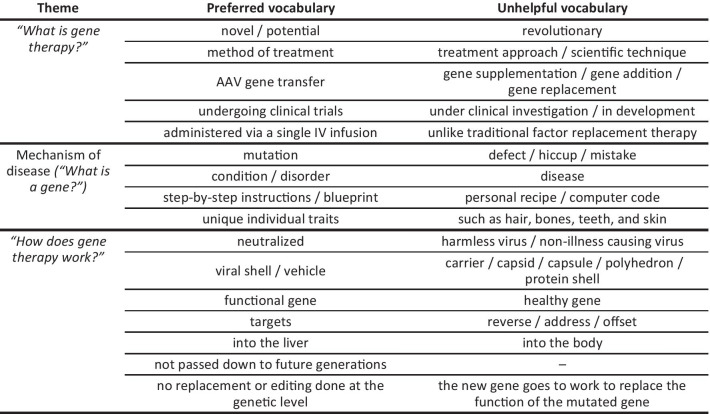
Summary of preferred versus unhelpful vocabulary for talking about gene therapy with hemophilia patients

**Fig. 4:**
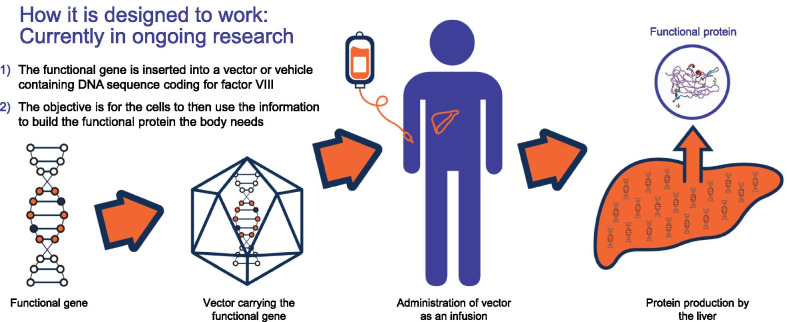
Pictorial summary that was agreed to best represent the concept of how gene therapy is designed to work
4. Preferred summary descriptor (how we describe it in one phrase): “Gene therapy is being studied in clinical trials with the aim to allow the body to produce factor VIII protein on its own”.

### Results by audience and country

**Fig. 5:**
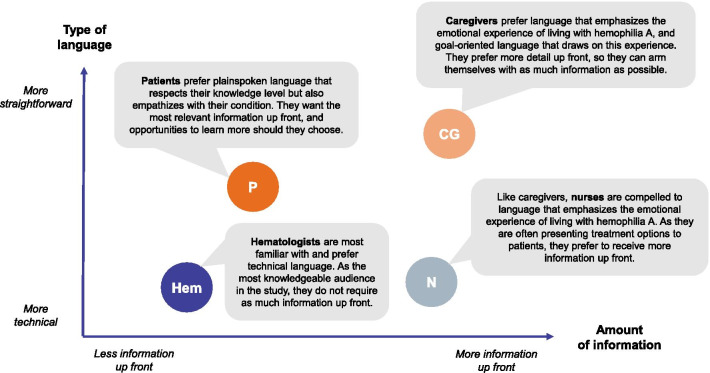
Key differences in language preferences of different audiences. CG, caregivers; Hem, hematologists; N, nurses; P, patients

Discussion of different story flow options in Phase III revealed differences in the type and amount of information relevant to different audiences (Fig. 5). As expected, HCPs are most familiar with technical language, while patients prefer straightforward language that respects that they have some knowledge about the subject, but without overwhelming them with scientific information.

Where differences between countries existed, country-specific recommendations were made. US and UK audiences preferred to describe hemophilia A as a “condition” or “disorder”, while “disease” was the accepted term in German, Italian, Spanish, and French, and was better understood than “condition” or “disorder”: “*Everyone understands what a disease is, but not everyone will understand disorder*”*,* “*disorder sounds light … less severe*”. For describing a gene, “blueprint” was well received by English-speaking audiences, whereas “step-by-step instructions” was the preferred term in European countries. There were differing levels of patient sensitivity to the word “mutation”; UK and French audiences were most sensitive to the term and preferred to use “variation” or “change”. Some words did not translate directly into another language; for example, a viral “shell” translates into “envelope” in French. The full recommended gene therapy lexicons for individual countries in local languages are presented in Additional File 3.

## Discussion

A recent review article considered perspectives of physicians and patients in discussing decisions to proceed with gene therapy for hemophilia and highlighted the need for sources of clear and reliable information to be able to discuss and judge the risks and benefits of potential new gene therapies [9]. Literature searches have failed to identify any studies focusing on language use among hemophilia communities relating either to disease management in general or to gene therapy. Therefore, we believe that this market research study is the first published study to report on the active development of a gene therapy lexicon. The outputs of the study identify a recommended language set for effectively communicating information about AAV-based gene therapy for hemophilia between and amongst stakeholders. Preferred words and phrases to describe the potential application of gene therapy in the treatment of hemophilia A were agreed under three main themes: *1. Mechanism of disease/What is a gene? 2. What is gene therapy? 3. How does gene therapy work?* It was also valuable to define words and phrases that participants considered unhelpful to include in conversations about gene therapy (see Table 4). An agreed summary narrative provides a concise, consistent and meaningful way to describe the concept of gene therapy and the potential of AAV gene therapy in the management of hemophilia A.

Conversations during the study showed that people with hemophilia are highly engaged and well educated about the condition and its treatment and are already very informed about the potential of gene therapy. These observations were not unexpected; patients and caregivers have dealt with their hemophilia for years and need to be competent at self-managing their condition [27]. According to a UK HCP in the study, “*Hemophilia A patients are very well-informed—they do a lot of research. They are fully capable of understanding what these terms mean.*”

The feedback received on the gene therapy language concepts was largely consistent across all audiences, allowing the development of a narrative that resonates across borders. However, there were some differences among audience types and countries. As expected, the type of language preferred by different audiences varied in terms of the amount of information provided and on a scale of straightforward/plain-spoken (for patients and caregivers) to more technical and detailed (for HCPs). Although HCPs may prefer to use more technical terms, they endorsed the language concepts designed for communicating with patients. When consistency was not possible due to cultural or language differences, country-specific recommendations were made (see Additional File 3).

Experience from the study also highlighted two key challenges for discussing gene therapy, which were adopted as guiding principles for developing the lexicon: (1) Providing the right amount of information: balancing the level of information required to fill gaps in patients’ existing knowledge versus overwhelming them with information; (2) Communicating with the right amount of emotion: being mindful of patient sensitivities versus accurately describing gene therapy. Conversations with people with hemophilia A showed that it is easy to lean too far one way and risk losing them or lean too far the other way and appear to not understand them at all. As an example, although patients may be alarmed by the word “virus”, it is important to talk about the role of AAV in gene transfer therapy up front*—*if not discussed until later, they may suspect that key information is being intentionally withheld, particularly given the legacy issues of transfusion-transmitted infections this community has experienced.

Effective communications are a fundamental component of shared clinical decision-making among patients and healthcare providers, a practice that has become especially important to people with hemophilia and has been redefined since the aforementioned scandal of contaminated factor replacement products in the 1980s and 1990s [28]. While several specific frameworks and decision support tools have been developed to underpin a more equal partnership between patients and doctors for decision-making about hemophilia management [23, 29–31], the agreement and use of a common vocabulary could further facilitate discussions about treatment choices and access, in particular relating to novel and complex treatment modalities currently being explored in clinical trials. In a qualitative study exploring factors that influence decisions about treatment among young men with hemophilia, the need for a common language to discuss treatment options was identified as a critical factor [24]. Ineffective communication between young men and their healthcare team risks a significant disconnect between patients and providers, and suboptimal knowledge about options for treatment [32].

Similar viewpoints were expressed by hematologists and patient advocacy group leaders involved in the planning phase of the study. Misconceptions and lack of understanding about gene therapy (among healthcare providers as well as patients), lack of accessible gene therapy education, and poor communication and engagement with young patients were all identified as barriers to informed treatment decision making. Common to these themes was the need for a shared language that reflects the voice of the patient, and the importance of developing a meaningful educational story flow rather focusing on facts alone.

The outputs of the present study are relevant for improving communications among the hemophilia community about the potential place of AAV gene therapy in the management of hemophilia and provide a basis for continued lexicon development as more evidence on the clinical outcomes of AAV gene therapy becomes available. The study also provides a model for engaging with patients and their care providers to develop agreed vocabularies in other fields in which the complexities of new treatment modalities can present a barrier to effective communications.

This study was a mostly qualitative evaluation of language concepts informed by expert opinion and experience and tested among a representative audience of people affected by hemophilia. Qualitative research is a valuable and structured methodology for evaluating data such as experiences, behaviors, preferences, and concepts that are not readily represented by numbers, and increasingly is being used to inform decisions about clinical practice and policy [33]. Specific guidelines exist to encourage transparent and consistent reporting of the conduct and findings of qualitative studies and have been followed in this paper [34]. Instant-response methodology, as used in our study, is an established, effective, and accurate way to understand which language resonates with a discussion group and how to refine it in a way that will be meaningful to similar audiences. Available technology for this method [25] allows a semiquantitative analysis of outputs, as presented in the results above.

Study participants were selected to represent the different stakeholders in the hemophilia community, and enrollment criteria designed to limit the proportions of individuals already actively involved with gene therapy or novel treatments and minimize potential bias were met. However, we acknowledge that their willingness to participate in the study could define them as individuals who are especially engaged and motivated members of this community, and this might be a limitation with regard to representation of the wider populations of hemophilia patients and care providers. The subjects who participated across countries provided generally consistent feedback that indicated a level of agreement on optimum language for communicating about gene therapy. A larger sample size would provide greater confidence in the results, and a follow-on study of the gene therapy lexicon involving more participants is already underway.

As clinical development of gene therapies for hemophilia continues, new data become available, and products receive approval, the preferred and accurate language will likely evolve. Objectives of the forthcoming follow-on study are to explore how the lexicon is being received in the hemophilia community; to test and refine the existing lexicon with more hematologists, nurses, patients, and caregivers in the US and Europe; and to address new topics of communication pertinent to the evolving status of gene therapy in hemophilia*.* While the focus of the present research was on finding the right language to convey information about the concept of AAV gene transfer, the next challenge will be finding the right way to discuss the results of gene therapy clinical trials to this highly informed, engaged, and individual audience. People with hemophilia are hungry for information on clinical efficacy, durability, and safety; and will also need to understand specific concepts about, for example, how long AAV gene transfer lasts, expected factor levels, any differences between *F8* and *F9* gene therapy, differences between different AAV platforms for the same gene transfer, data relating to potential integration events, and what happens if the one-time infusion does not work. Finally, although participants in the study were chosen mainly for their experiences relating to hemophilia A, the research approach was focused on general gene therapy language concepts and we therefore anticipate that the learnings could also be applied to the hemophilia B community.

## Conclusions

People living with hemophilia are generally well informed about hemophilia and available treatments and consequently are highly engaged in managing their condition and learning about novel treatment options. Similarly, HCPs and caregivers involved in the management of hemophilia are highly engaged and informed about the condition and current or potential treatment options. By using accurate and straightforward language, the community can readily understand the complex concept of AAV gene therapy for hemophilia A and how it differs from factor replacement therapy or other currently available treatments. Gene therapy is best understood by patients as the transfer, via a single, one-time IV infusion, of a functional gene inserted into a neutralized viral shell, which delivers the new gene into the liver, with the aim to allow the body to produce factor VIII protein on its own. Knowing that this is not gene replacement or gene editing, and that the transferred gene is not passed down to future generations, is also important to patients.

In summary, this study suggests that consistent use of hemophilia community-informed lexicon among HCPs, caregivers, and patients can minimize miscommunication while also facilitating conversations and informed decision-making regarding potential future treatment opportunities and choices. Further gene therapy lexicon development for the hemophilia community will be needed and is underway, with a focus on additional concepts and findings as this field continues to evolve rapidly and educational needs increase.

## Supplementary information

**Additional File 1**. Baseline language and images describing gene therapy for hemophilia A audiences used in Phase II focus group discussions.

**Additional File 2**. Story flow options describing gene therapy for hemophilia A audiences used in Phase III focus group discussions.

**Additional File 3**. Country-specific versions of the recommended gene therapy lexicon: Summary of preferred vocabulary for talking about gene therapy with hemophilia patients (French, German, Italian, Spanish).

## Availability of data and materials

Data sharing is not applicable to this article as no datasets were generated or analyzed during the current study.
