# Supplementary material for: Optimizing language for effective communication of gene therapy concepts with hemophilia patients: a qualitative study
Source: Orphanet J Rare Dis. 2021 Apr 28;16:189. doi: 10.1186/s13023-020-01555-w (PMC8082836; doi:10.1186/s13023-020-01555-w)
Supplement: Supplementary file 1 — Additional File 1. Baseline language and images describing gene therapy for hemophilia A audiences used in Phase II focus group discussions. [file 13023_2020_1555_MOESM1_ESM.pdf]

Additional File 1 Baseline language and images describing gene therapy for hemophilia A audiences used in Phase II focus group discussions.

THEME	BASELINE LANGUAGE AND IMAGE CONCEPTS FOR DISCUSSION (PHASE II)
**What is a gene?**	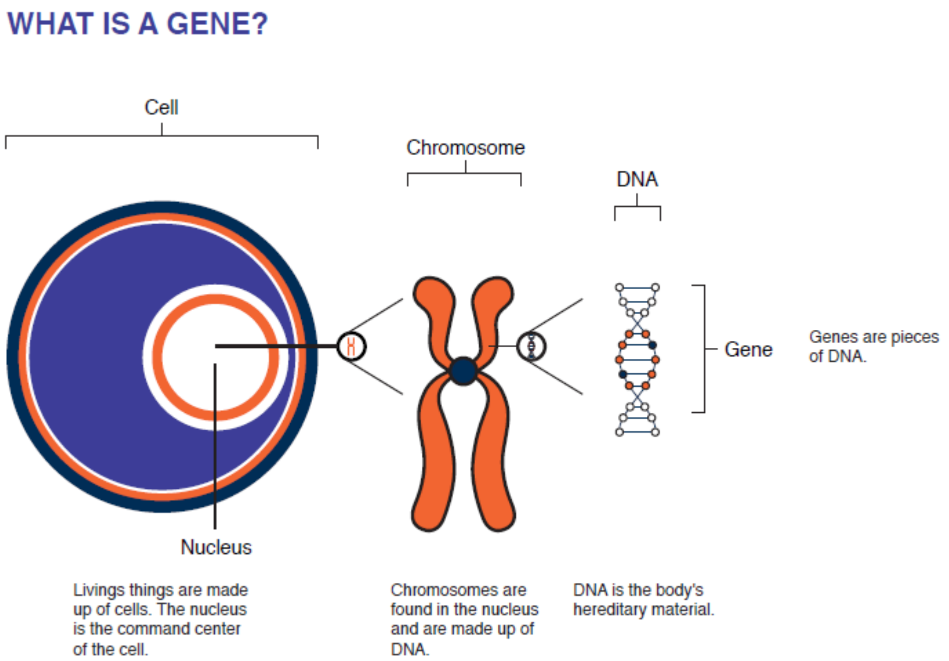
	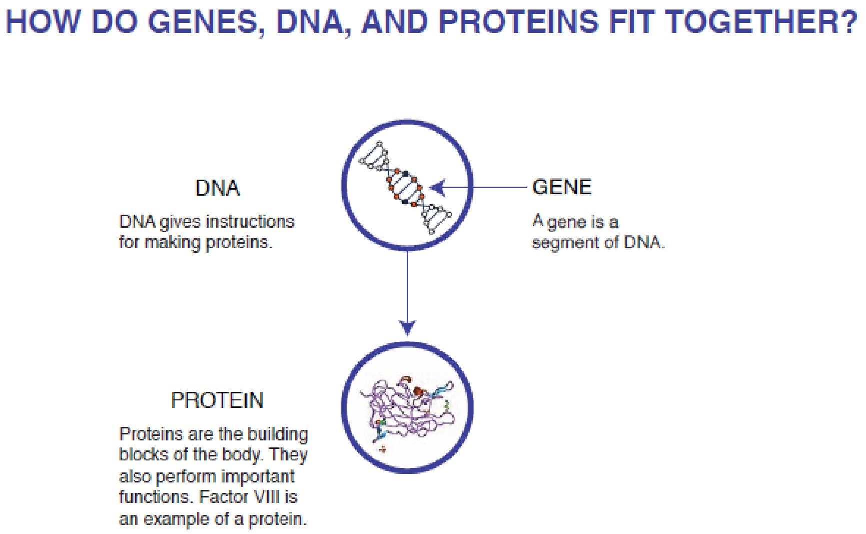
	1. In your body, genes provide the **step-by-step instructions** for making proteins, like Factor VIII, that your body needs to run properly.<br>2. A gene is the **blueprint** for constructing your body's unique individual traits such as bones, teeth, muscles, skin, hair and blood, as well as proteins that help your body function, such as Factor VIII.<br>3. Genes are the **key to your unique genetic makeup**. Genes contain DNA, which is the **code that makes you, you**.<br>4. Genes contain DNA – your personal **recipe** for making each individual part of the body.

THEME	BASELINE LANGUAGE AND IMAGE CONCEPTS FOR DISCUSSION (PHASE II)
**Mechanism of disease**	**Mechanism of genetic diseases**<br>• We're all born with about **20,000 genes**. Like the code in a computer, these genes are the information that tell our bodies how to function. And nearly all of these genes are the same in everyone.<br>• Occasionally, there's a blip in the code. A **piece of DNA** making up the gene may be **missing, reversed, or duplicated**. This variance in a single gene, also known as a **mutation**, can cause a slight change in how our body operates. **Just like a computer program**, this genetic hiccup can cause certain functions of our body to not work as intended.<br>• Many of these variations are harmless and go undetected. But when they cause symptoms or illness, these **genetic variances** are what are broadly referred to as **genetic diseases**. They are either inherited at birth in the genes we receive from our parents, or they can occur spontaneously during a person's life. Whether new or inherited, the effects of **genetic disorders** are the same.<br>**Mechanism of hemophilia A**<br>• Genetic diseases are caused by an **irregularity in our genetic makeup**. These irregularities, known as mutations, can make it so that certain genes don't function the way they should.<br>• Hemophilia A is a genetic disease caused by a mutation in the gene responsible for making the protein our bodies rely on to form stable clots in our blood, Factor VIII. Our bodies need a certain level of Factor VIII to stop prolonged bleeding, particularly inside joints and muscles.<br>• Hemophilia A **primarily impacts males**, as the gene responsible for producing Factor VIII is located on the X chromosome. Because males have one X and one Y chromosome, if they either inherit a hemophilic gene or a mutation occurs spontaneously, they develop the condition. Females have two X chromosomes and are therefore less likely to be affected by the disease themselves – but can carry the mutated gene and pass it on to their children, making them carriers of the disease. In some cases, they too can have symptoms of the disease.
	**The language of mutation**<br>1. a change in a gene's DNA sequence.<br>2. a genetic hiccup.<br>3. a gene mutation.<br>4. a variation in your genes.<br>5. a mistake in your genetic sequence.<br>6. a genetic defect.<br>7. a variance in the genetic code.
**What is gene therapy?**	1. Gene therapy is a **scientific technique** that works to **offset the underlying genetic defect** in patients who suffer from genetic diseases.<br>2. Gene therapy is a **potential method** of treatment for Hemophilia A patients. Unlike traditional factor replacement therapies, gene therapy works **from the inside out**, **targeting the underlying genetic defect** that causes Hemophilia A.<br>3. Gene therapy is a potential mode of treatment for patients who have genetic diseases like Hemophilia A. Gene therapy as a treatment option is still undergoing clinical trials but **shows promise** for Hemophilia A patients.<br>4. Gene therapy is a **treatment approach** currently **in development** that works to **address the genetic variation** that causes Hemophilia A.<br>5. Gene therapy is a medical approach currently under **clinical investigation**. Gene therapy is **not a band-aid approach** as it is designed to **reverse the underlying genetic defect** in patients that suffer from genetic diseases, like Hemophilia A.<br>6. Gene therapy is a form of treatment that involves **inserting genetic information** in the form of DNA into cells.<br>7. Gene therapy is a **novel treatment approach** where **genetic material** is used to treat a genetic disease **at its source**.<br>8. Gene therapy is **different from traditional** Factor VIII replacement **therapy**. It **targets the root cause** of genetic disease to **treat** patients with Hemophilia A.

<table>
<tr><th>THEME</th><th>BASELINE LANGUAGE AND IMAGE CONCEPTS FOR DISCUSSION (PHASE II)</th></tr>
<tr><td>What is gene therapy?

Continued</td><td>Images that align with the concept of “gene therapy”
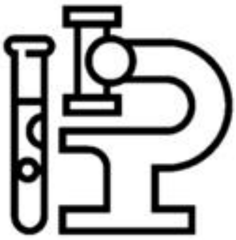
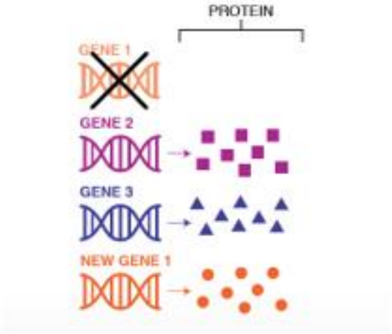

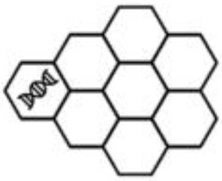
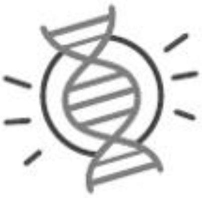
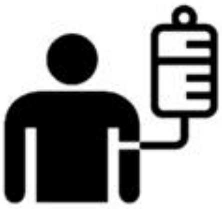
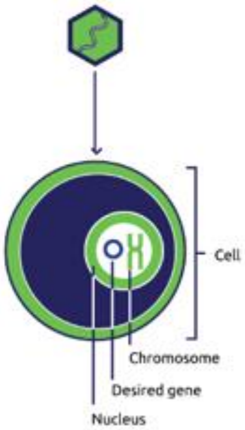

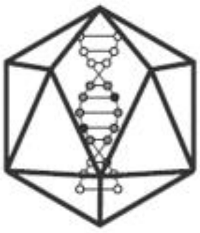
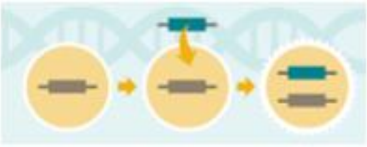
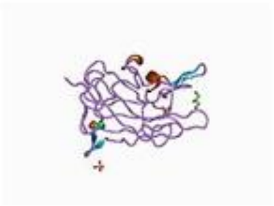</td></tr>
<tr><td>How does gene therapy work?</td><td>Gene therapy for Hemophilia A
Gene therapy has shown promising signs for many years in a variety of diseases and disorders, including Hemophilia A. One method of gene therapy that is currently in clinical trials for Hemophilia A is called gene transfer. In this method, a Factor VIII gene is inserted into a protein shell, which is then administered to a patient via infusion.
Once it has been infused, the functional gene works alongside the one that causes the Factor VIII deficiency of Hemophilia A. That means there is no replacement or editing done at a genetic level. The new gene is simply added, allowing it to play the role that the original gene was intended to – without the variation that causes Hemophilia A. Ultimately, gene therapy makes it possible for the patient’s body to produce Factor VIII at a level required to manage Hemophilia A.</td></tr>
<tr><td></td><td>Naming gene therapy
Adeno-associated virus (AAV) gene therapy
Gene transfer
Gene addition
Gene supplementation
5. Gene replacement</td></tr>
</table>

THEME	BASELINE LANGUAGE AND IMAGE CONCEPTS FOR DISCUSSION (PHASE II)
**How does gene therapy work?**<br><br>*Continued*	**In vivo and ex vivo gene therapy for genetic diseases (FOR HCPs ONLY)**<br>• There are many different types of gene therapy under investigation for a variety of diseases and disorders. And all of these gene therapies involve one of two different methods – **in vivo** and **ex vivo**. In in vivo gene therapy, a **functional gene** is inserted into a **vehicle** called a vector, which is then injected or infused into a patient.<br>• In ex vivo gene therapy, this process happens **outside the body**. First, **affected cells** are removed from the body via a **biopsy**. Functional genetic material is then inserted into a vector, and administered to the harvested cells. The cells undergo a change in this process, and then are transplanted back into the patient's body.<br>• In both of these methods, the functional genetic material packaged into the vector works to **produce therapeutic proteins locally**. That means gene therapy has the potential to provide a **long-term durable benefit** for patients with genetic diseases.
	**Types of gene therapy (FOR HCPs ONLY)**<br>1. Gene transfer<br>2. Gene editing<br>3. Cell therapy<br><br>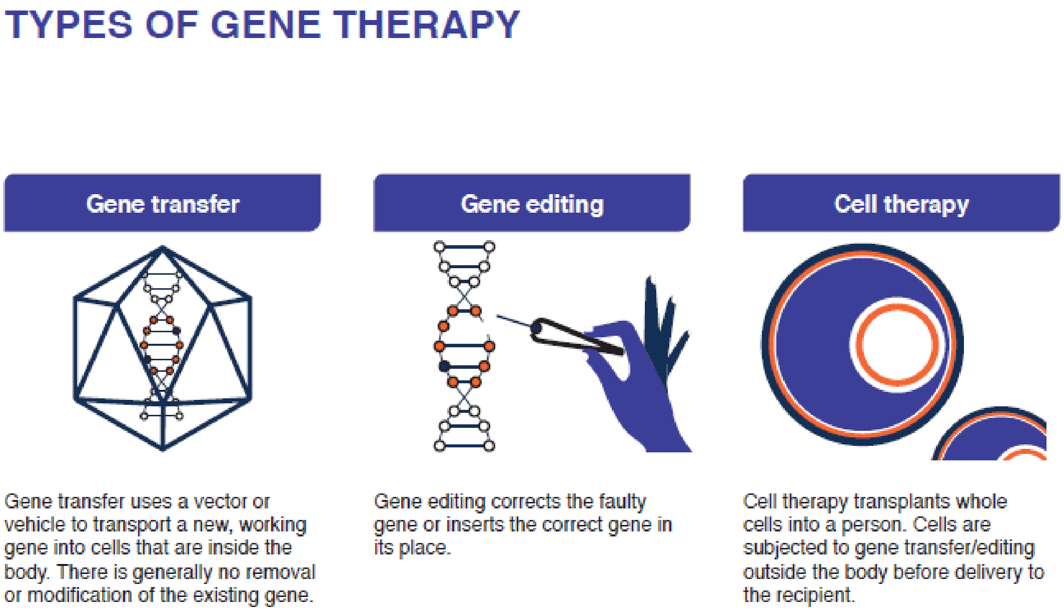

**THEME**	**BASELINE LANGUAGE AND IMAGE CONCEPTS FOR DISCUSSION (PHASE II)**
**How does gene therapy work?** *Continued*	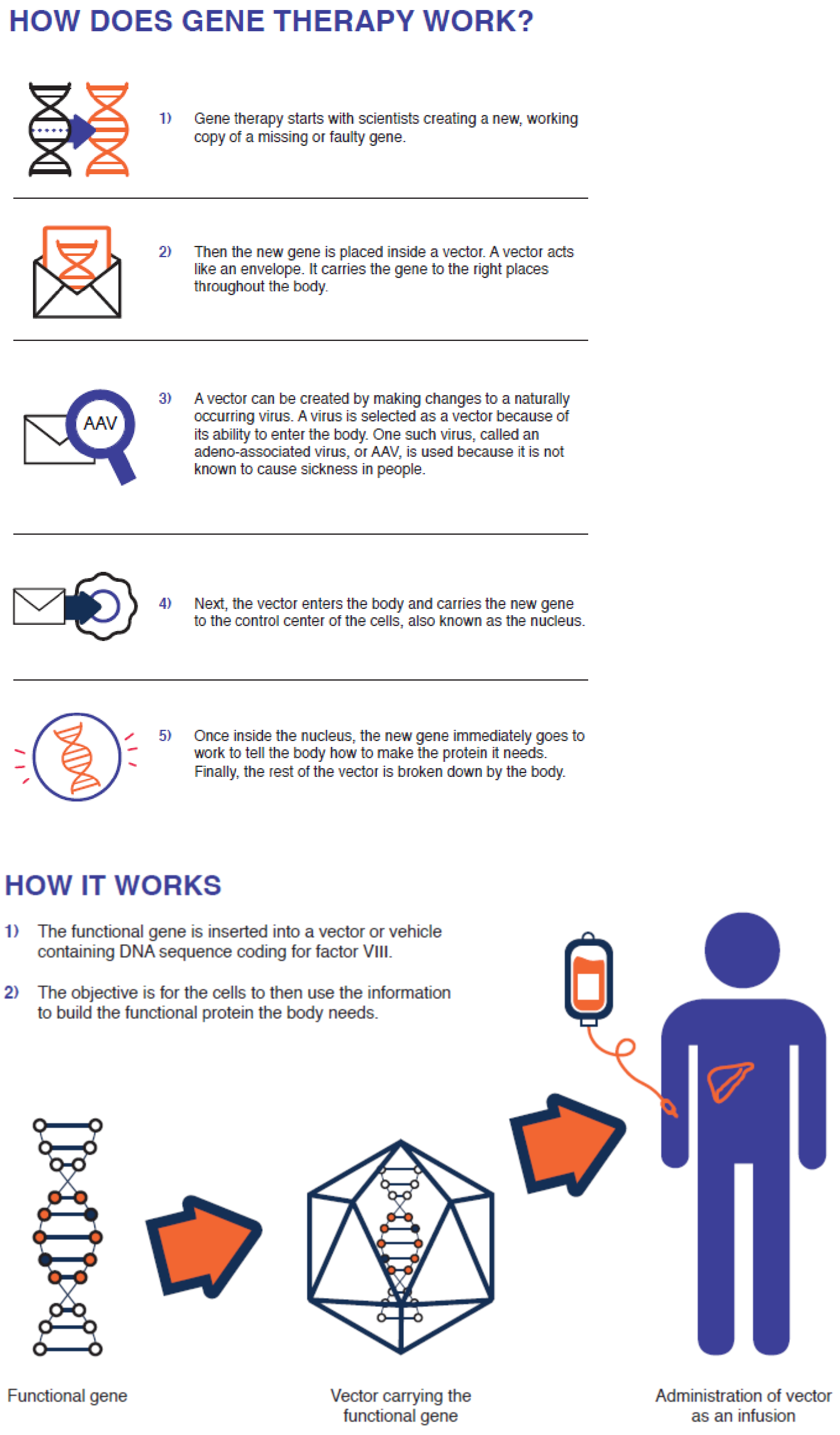

THEME	BASELINE LANGUAGE AND IMAGE CONCEPTS FOR DISCUSSION (PHASE II)
**How does gene therapy work?**<br><br>*Continued*	**What a viral vector is**<br>1. a carrier<br>2. an envelope<br>3. a vehicle<br>4. a protein shell<br>5. a capsid<br>6. a polyhedron<br>7. a container<br>8. a capsule<br>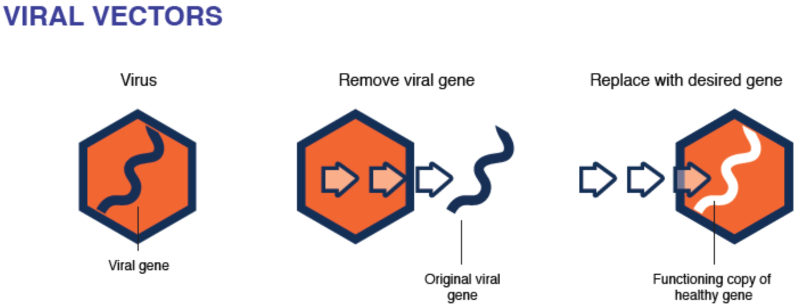
	**AAV is a harmless virus**<br>1. a nonpathogenic virus<br>2. a helper virus<br>3. a friendly virus<br>4. a harmless virus<br>5. a viral shell<br>6. a non-illness causing virus<br>7. a virus with its viral genetic material removed<br>8. a virus that has been neutralized<br>9. a virus that has been made safe
**Goals of gene therapy**	**Therapeutic goals of gene therapy**<br>• For many years, the primary way to manage Hemophilia A has been through Factor VIII replacement therapy. This can help manage and prevent symptoms, and for many patients, offers a significant therapeutic benefit. But for many others there is still an **unmet need**.<br>• Gene therapy for Hemophilia A has the potential to meet that unmet need. One type of gene therapy that is **specifically designed for Hemophilia A patients** – the **first of its kind** for patients with this disease – is currently undergoing clinical trials. And the initial results are promising. Unlike traditional Factor VIII replacement therapy, gene therapy provides patients with the genetic code that may allow their bodies to produce and achieve a new, higher baseline level of Factor VIII. This has the **potential to reduce or eliminate** the need for replacement therapy – both prophylactically and on demand.<br>• The **durability of this treatment is still under investigation**. But the prospect that this treatment could allow severe Hemophilia A patients to produce their own Factor VIII, and not be dependent on Factor VIII replacement therapy – something they have never been able to do before – is truly **revolutionary**.<br><br>**Goals of gene therapy broadly**<br>• Gene therapy has the potential to **provide a benefit for Hemophilia A patients that Factor VIII replacement therapy could not** do. With gene therapy, Hemophilia A patients may have the ability to **produce their own Factor VIII**.<br>• But gene therapy offers more than a simple therapeutic benefit. It means the possibility of spending less time treating with prophylaxis, and more time living life. That could mean not having to spend time every day giving injections. For others, it means **moving forward with daily activities without the added burden of worrying about spontaneous bleeds** – or worrying about the complications that come with those bleeds. In short, it could mean **more freedom, and less worry** for patients and their families.<br>• **Empowering patients** and their loved ones to live life without the constant weight that Hemophilia A puts on their shoulders – that's what gene therapy for Hemophilia A patients is all about.
