# Supplementary material for: Optimizing language for effective communication of gene therapy concepts with hemophilia patients: a qualitative study
Source: Orphanet J Rare Dis. 2021 Apr 28;16:189. doi: 10.1186/s13023-020-01555-w (PMC8082836; doi:10.1186/s13023-020-01555-w)
Supplement: Supplementary file 2 — Additional File 2. Story flow options describing gene therapy for hemophilia A audiences used in Phase III focus group discussions. [file 13023_2020_1555_MOESM2_ESM.pdf]

Additional File 2 Story flow options describing gene therapy for hemophilia A audiences used in Phase III focus group discussions.

<table>
<tr><th>THEME</th><th>STORY FLOW OPTIONS FOR DISCUSSION (PHASE III)</th></tr>
<tr><td>Story Flow Option A – Right to the Point</td><td>

What is gene therapy? → How does gene therapy work? → Goals of gene therapy

- Gene therapy is a **potential method of treatment currently undergoing clinical trials** for a variety of genetic conditions, including hemophilia A. Unlike traditional factor replacement therapies, gene therapy works **from the inside out, targeting** the mutated gene that causes reduced factor VIII production.
- The type of gene therapy used for hemophilia A patients is called **gene transfer**, which is administered via a single IV infusion. **This treatment allows the body's own cells to produce Factor VIII**.
- Gene transfer has the potential to help patients live life to the fullest. This means moving forward with daily activities without the burden of worrying about spontaneous bleeds, the complications of those bleeds, and the factor replacement needed to treat them. In short, gene transfer could mean more freedom, and less worry for patients and their families.

</td></tr>
<tr><td></td><td>

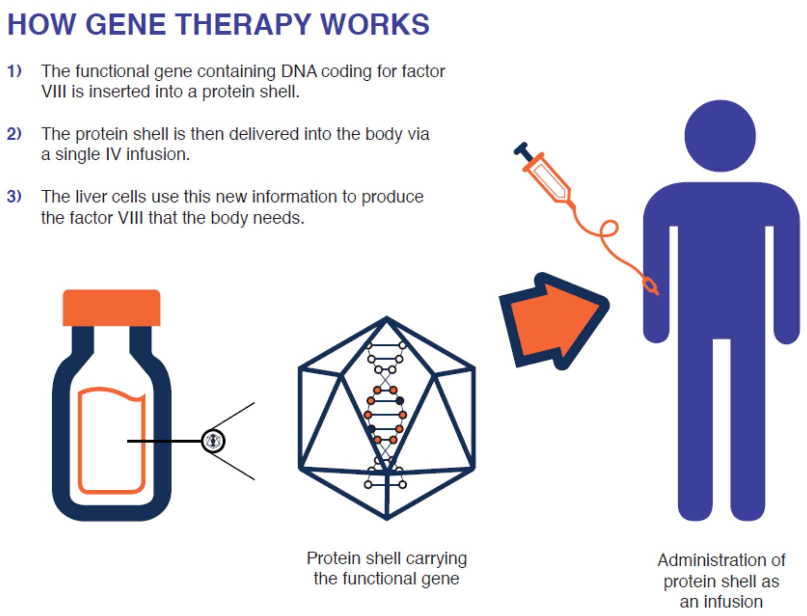

- Use of double helix or single-stranded DNA?

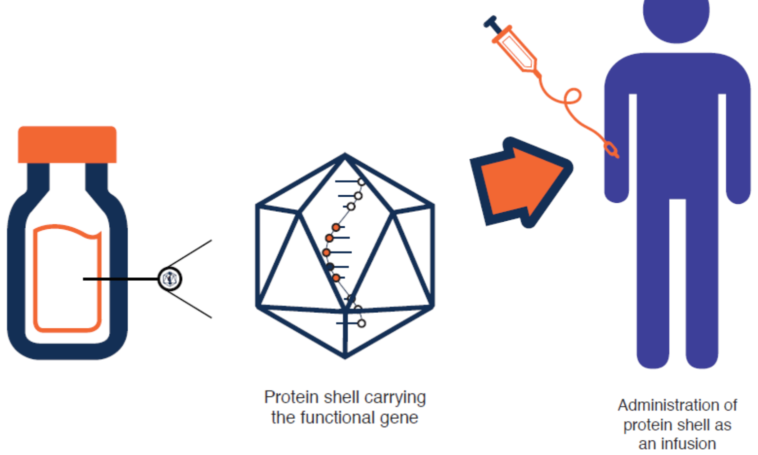

</td></tr>
</table>

THEME	STORY FLOW OPTIONS FOR DISCUSSION (PHASE III)
**Story Flow Option B – The Middle Ground Approach**	**What is gene therapy? → Mechanism of disease/ Why hemophilia A? → How does gene therapy work? → Goals of gene therapy**<br>• Gene therapy is a novel **treatment approach** in which genetic material is used to treat the biological basics of hemophilia A.<br>• The method of gene therapy that is currently **in clinical trials** for hemophilia A is called Adeno-**Associated Virus (AAV) gene therapy**. Because of a **genetic variance**, people with hemophilia A don't produce enough of the Factor VIII protein, which is necessary to form stable clots in their blood. AAV gene therapy targets the gene responsible for creating Factor VIII.<br>• In AAV gene therapy, **a functional gene** is inserted into a **nonpathogenic virus** which acts as an **envelope** to deliver the new gene into the body via a single IV infusion. There is **no replacement or editing done at a genetic level** – just the introduction of a new, functional factor VIII gene into the body, which is not passed down to future generations.<br>• The goal of this treatment is to provide patients with the ability to produce and maintain a higher baseline level of factor VIII.
	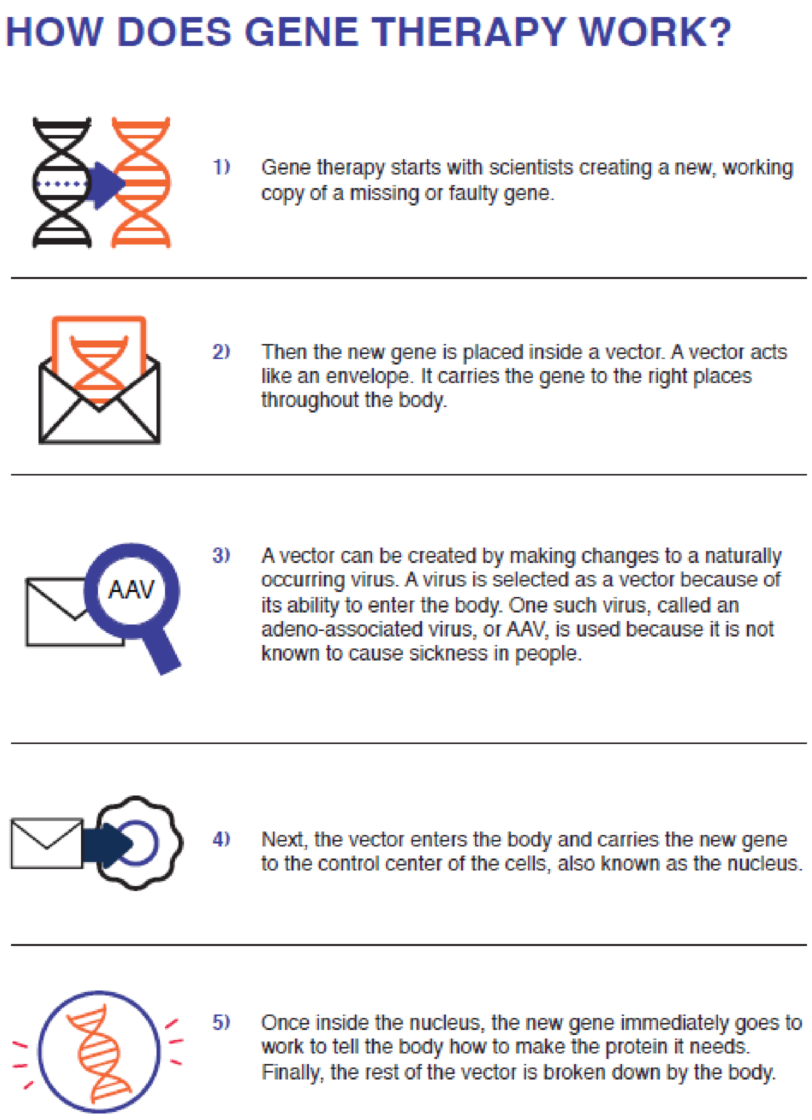

THEME	STORY FLOW OPTIONS FOR DISCUSSION (PHASE III)
**Story Flow Option C – Leading with Goals**	**Goals of gene therapy → What is a gene? → Mechanism of disease → What is gene therapy? → How does gene therapy work?**<br>• Empowering **you** and your loved ones to live life without the constant weight hemophilia A puts on your shoulders is what gene therapy is all about.<br>• In your body, genes are pieces of DNA, which provide the **step-by-step instructions** for making proteins like factor VIII. **Hemophilia A patients** inherit or spontaneously develop a **genetic mutation** that inhibits their bodies' ability to make factor VIII – the protein that allows blood to clot.<br>• Gene therapy is a **revolutionary treatment approach** for patients like you, and is **currently undergoing clinical trials**. In this approach, a **working** gene is inserted into a **neutralized viral shell**, which is then delivered into your body via a one-time IV infusion. **No genes are removed or replaced in this process – the new gene is simply added** to do the extra work the original gene that causes the **genetic disorder (disease)** could not do.<br>• The goal is that the new gene provides the genetic information necessary to produce factor VIII at a level you need to live your life free from the emotional and physical burden that comes with having hemophilia A.
	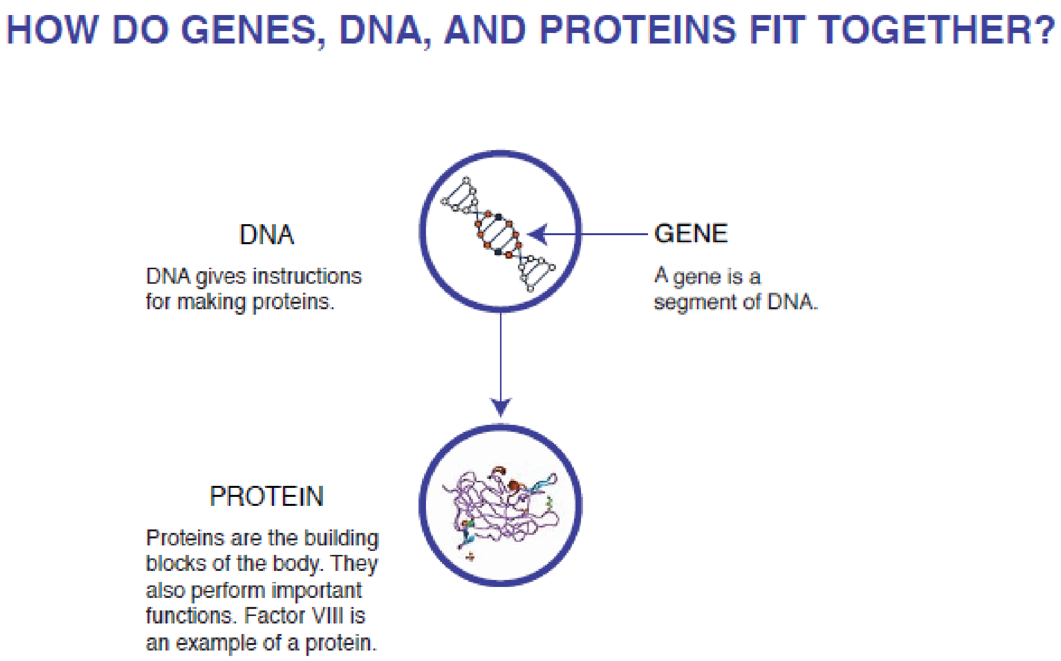

THEME	STORY FLOW OPTIONS FOR DISCUSSION (PHASE III)
**Story Flow Option D – Liver Story**	**Mechanism of disease → What is gene therapy? → How does gene therapy work? → Goals of gene therapy**<br>• In a person without hemophilia A, genes in the liver and other parts of the body provide the instructions needed to produce Factor VIII, a protein needed for clotting. But in a person with hemophilia A, a **genetic mutation** makes it so that not enough Factor VIII is produced.<br>• That's where gene therapy comes in. Gene therapy is **a new kind of treatment** for hemophilia A. **Unlike traditional factor replacement therapy**, it doesn't just replace the missing factor. It works to **restore cellular function**, allowing them to produce Factor VIII.<br>• The type of gene therapy **that may soon be available** for hemophilia A patients is called **gene transfer**. In gene transfer, a new, functional gene is inserted into a **protein shell**, which is then administered to the patient in a single IV infusion. This protective shell, which is made from a **neutralized virus**, carries the functional genetic material into the liver. In the liver, the new gene goes to work to **replace the function** of the mutated gene.<br>• Ultimately, this process allows the liver to produce Factor VIII itself. That means the potential for no more prophylactic or on-demand factor replacement, and a life **post-hemophilia**.
**Story Flow Option E – Original Flow**	**What is a gene? → Mechanism of disease → What is gene therapy? → How does gene therapy work? → Goals of gene therapy**<br>• A gene is the **blueprint** for constructing your body's unique individual traits such as bones, teeth, muscles, skin, hair and blood, as well as proteins that help your body function, such as factor VIII.<br>• A variation in a gene can be harmless. However, **some changes in a gene's DNA sequenc**e can cause **genetic conditions** like hemophilia A. Hemophilia A is caused by a **mutation** in the factor VIII gene, which impedes production of this key clotting factor in the blood.<br>• For many years, the primary way to manage hemophilia A has been through factor VIII replacement therapy – either prophylactic, on-demand, or both. Replacement therapy helps manage and prevent symptoms. But for many patients there is still an unmet need.<br>• Unlike traditional replacement therapies, **gene transfer targets the root cause of hemophilia A**. A new, functional gene is inserted into a **vehicle** made from a **non-illness causing virus**, which is delivered into the patient via a single IV infusion.<br>• While still **undergoing clinical trials**, gene transfer has the potential to allow severe hemophilia A patients with the ability to produce their own factor VIII – something they have never been able to do before.
	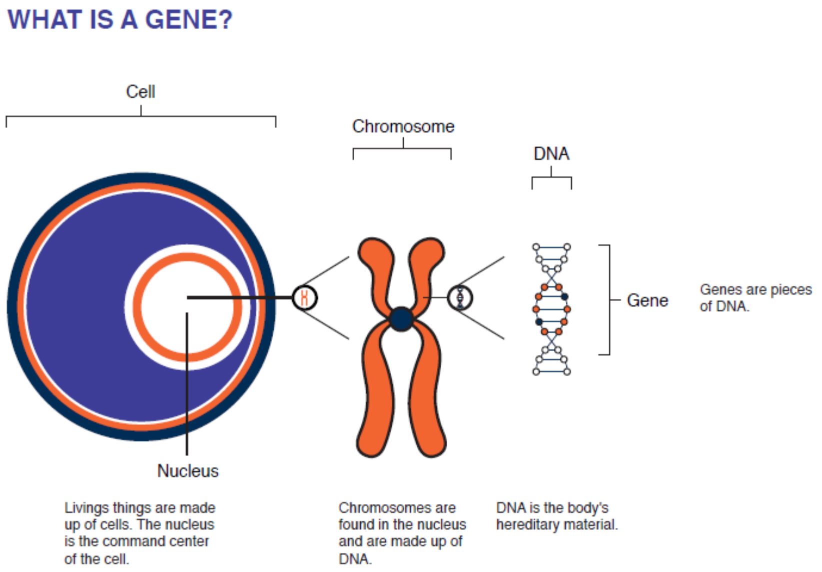

<table>
<tr><th>THEME</th><th>ADDITIONAL LANGUAGE AND IMAGE CONCEPTS FOR DISCUSSION (PHASE III)</th></tr>
<tr><td>The language of “mutation”</td><td>

Hemophilia A is caused by…

1. a change in a gene’s DNA sequence.
2. a gene mutation.
3. a variation in a single gene.
4. a variance in the genetic code

</td></tr>
<tr><td>How to explain gene therapy</td><td>

Gene therapy…

1. works from the inside out.
2. treats hemophilia A at its source.
3. targets the root cause of hemophilia A.
4. treats the biological basis of hemophilia A

</td></tr>
<tr><td>How to explain gene transfer, specifically</td><td>

In gene transfer…

1. the new gene begins producing Factor VIII
2. there is no replacement or editing done at a genetic level – just the introduction of a new, functional factor VIII gene into the body.
3. no genes are removed or replaced in this process – the new gene is simply added to do the extra work the original gene that causes the genetic disorder could not do.
4. the new gene goes to work to replace the function of the mutated gene.

</td></tr>
<tr><td>Describing what a vector is</td><td>

When a hemophilia A patient undergoes gene therapy, the new gene is inserted into _______________ (otherwise called a vector) which is inserted directly into the body via a single infusion.

1. a carrier
2. an envelope
3. a vehicle
4. a protein shell
5. a protective shell
6. a viral shell
7. a viral envelope
8. an adeno-associated virus (AAV)

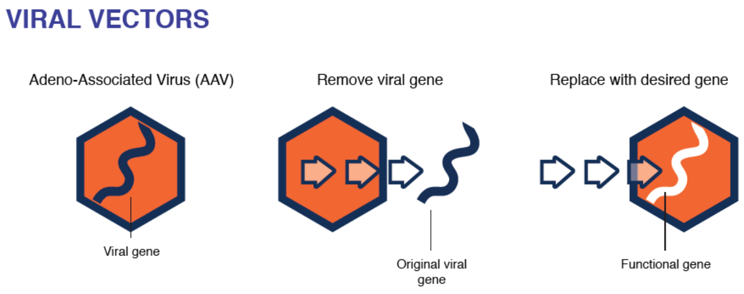

</td></tr>
<tr><td>Assuaging fears when it comes to the adeno-associated “virus”</td><td>

When a hemophilia A patient undergoes gene therapy, _______________________, provides the container that houses the new gene.

1. a nonpathogenic virus
2. a non-illness causing virus
3. a neutralized virus
4. an altered virus
5. a modified virus

</td></tr>
</table>
