# Supplementary material for: Optimizing language for effective communication of gene therapy concepts with hemophilia patients: a qualitative study
Source: Orphanet J Rare Dis. 2021 Apr 28;16:189. doi: 10.1186/s13023-020-01555-w (PMC8082836; doi:10.1186/s13023-020-01555-w)
Supplement: Supplementary file 3 — Additional File 3. Country-specific versions of the recommended gene therapy lexicon: Summary of preferred vocabulary for talking about gene therapy with hemophilia patients (French, German, Italian, Spanish). [file 13023_2020_1555_MOESM3_ESM.pdf]

Additional File 3 Country-specific versions of the recommended gene therapy lexicon: Summary of preferred vocabulary for talking about gene therapy with hemophilia patients (French, German, Italian, Spanish)

FRANCE

	**+ Langue à utiliser**	
Qu'est-ce que la thérapie génique?	novel	potentielle
	une méthode de traitement	
	transfert de gènes AAV	
	en études cliniques	
	administré par une seule perfusion IV	
Mécanisme de la maladie (Qu'est-ce qu'un gène?)	mutation	
	maladie	
	les instructions étape-par-étape	
	caractéristiques uniques	
Comment fonctionne la thérapie génique?	neutralisée	
	une enveloppe virale	véhicule
	gène fonctionnel	
	cible	
	dans le foie	
	n'est pas transmis aux générations futures	
	aucun remplacement ou édition génétique n'est effectué	

GERMANY

	**+ Sprachgebrauch**	
Was ist eine Gentherapie?	neuartiger	potenziell
	Therapiemethode	
	AAV Gentransfer	
	aktuell in klinischer Erprobung befinden	
	mittels einer einzigen intravenösen Infusion transportiert	
Krankheitsmechanismus (Was ist ein Gen?)	mutation	
	erkrankung	
	schrittweise Anleitungen	
	der einzigartigen Eigenschaften	
Wie funktioniert eine Gentherapie?	neutralisierter	
	virale Hülle	Vehikel
	funktionierendes Gen	
	zielt	
	in die Leber	
	nicht an nachfolgende Generationen weitergegeben	
	findet auf der genetischen Ebene keine genetische Ersetzung und kein Gen-Editing statt	

ITALY

	**+ Lingua da usare**	
Cos'è la terapia genica?	nuovo	potenziale
	metodo di trattamento	
	trasferimento genico AAV	
	attualmente in fase di sperimentazione clinica	
	viene somministrato tramite una singola infusione per via endovenosa	
Meccanismo di difesa (Cos'è un gene?)	mutazione	
	malattie	
	istruzioni passo passo	
	caratteristiche individuali uniche	
Come funziona la terapia genica?	neutralizzato	
	guscio virale	veicolo
	gene funzionale	
	prende di mira	
	Nel fegato	
	che non sitrasmesso ereditariamente	
	non sostituzione o modifica a livello genetico	

SPAIN

	**+ Terminología a utilizer**	
¿Qué es el tratamiento génico?	novedoso	potencial
	método de tratamiento	
	transferencia génica mediante AAV	
	en ensayos clínicos	
	se administra mediante una única infusión IV	
Mecanismo de la enfermedad (Qué es un gen?)	mutación	
	enfermedad	
	instrucciones paso por paso	
	rasgos únicos	
¿Cómo funciona el tratamiento génico?	neutralizado	
	capa vírica	vehículo
	gen funcional	
	dirige	
	hasta el hígado	
	que no se transmite a las siguientes generaciones	
	ninguna sustitución, ni edición a nivel genético	
